# Estimation of a biofilm-specific reaction rate: kinetics of bacterial urea hydrolysis in a biofilm

**DOI:** 10.1038/npjbiofilms.2015.14

**Published:** 2015-09-16

**Authors:** James M Connolly, Benjamin Jackson, Adam P Rothman, Isaac Klapper, Robin Gerlach

**Affiliations:** 1 Center for Biofilm Engineering, Montana State University, Bozeman, MT, USA; 2 Department of Chemical and Biological Engineering, Montana State University, Bozeman, MT, USA; 3 Department of Mathematical Sciences, Montana State University, Bozeman, MT, USA; 4 Department of Mathematics, Temple University, Philadelphia, PA, USA

## Abstract

**Background/Objectives:**

Biofilms and specifically urea-hydrolysing biofilms are of interest to the medical community (for example, urinary tract infections), scientists and engineers (for example, microbially induced carbonate precipitation). To appropriately model these systems, biofilm-specific reaction rates are required. A simple method for determining biofilm-specific reaction rates is described and applied to a urea-hydrolysing biofilm.

**Methods:**

Biofilms were grown in small silicon tubes and influent and effluent urea concentrations were determined. Immediately after sampling, the tubes were thin sectioned to estimate the biofilm thickness profile along the length of the tube. Urea concentration and biofilm thickness data were used to construct an inverse model for the estimation of the urea hydrolysis rate.

**Results/Conclusions:**

It was found that urea hydrolysis in *Escherichia coli* MJK2 biofilms is well approximated by first-order kinetics between urea concentrations of 0.003 and 0.221 mol/l (0.186 and 13.3 g/l). The first-order rate coefficient (*k*_1_) was estimated to be 23.2±6.2 h^−1^. It was also determined that advection dominated the experimental system rather than diffusion, and that urea hydrolysis within the biofilms was not limited by diffusive transport. Beyond the specific urea-hydrolysing biofilm discussed in this work, the method has the potential for wide application in cases where biofilm-specific rates must be determined.

## INTRODUCTION

The hydrolysis of urea by microorganisms has been widely shown as an effective method for the *in situ* production of alkalinity and the subsequent precipitation of carbonate minerals. At circumneutral pH, urea (CO(NH_2_)_2_) hydrolysis can be written as

(1)CO(NH2)2+2H2O+H+→2NH4++HCO3−,

where two ammonium ions and one bicarbonate ion are formed for each urea molecule that is hydrolysed. In addition, one proton is consumed which raises the pH. When calcium or other divalent cations are present carbonate mineral precipitation can be possible due to an increase in carbonate (CO_3_^2−^) concentration.

Carbonate mineral formation through microbial ureolysis is significant in medicine because of the role that ureolytic microorganisms can have in the formation of kidney and urinary tract stones as well as catheter encrustations.^1,2^ Mineral formation through ureolysis has also been studied extensively for engineered applications in building materials^3^ and in the subsurface for permeability reduction, soil stabilisation and contaminant remediation.^4^ Microbial urea hydrolysis is also of interest in industrial and agricultural wastewater treatment settings where a significant portion of the total nitrogen is attributed to urea.^5^ In all the systems mentioned above, particularly where the surface area to volume ratio is high, a significant portion of the ureolytic activity is likely to be attributed to biofilms.

A significant obstacle in predicting system behaviour in biofilm-containing systems is the lack of quantitative, system-specific reactive transport characterisation. This study looks specifically at ureolysis in a model biofilm system to shed light on the reactive transport environment that is likely to be present in systems that have, or have been stimulated to have, ureolytic activity. Ureolysis in planktonic cultures has been investigated thoroughly and there have been a number of studies to quantify volume-averaged ureolysis rates in porous media containing microorganisms^6–9^ and immobilised enzyme.^10,11^ The most applicable studies concentrate on the rate of precipitation in these systems leaving the micro-scale reactive transport characteristics of ureolysis in biofilms largely undiscussed.

Rather than taking a volume-averaged approach, ureolytic biofilms were grown in silicon tubes that mimic the laminar flow environment that would be encountered in a soil pore or urinary tract. No calcium or other cations that would cause mineral precipitation were supplied to the system so that urea hydrolysis could be studied in detail without the complications introduced when precipitation takes place. Precipitation has the potential to impact urea hydrolysis kinetics but was not investigated in this study. The silicon tubes were destructively sampled and thin sectioned to obtain an accurate biofilm thickness profile over the length of the tube. Biofilm thickness profiles were combined with influent and effluent urea measurements to obtain a ureolysis rate per biofilm volume (here referred to as a biofilm-specific rate). This paper provides a method for the measurement of effective reaction rates in biofilms and greater insight into the reactive transport environment of biofilm-catalysed urea hydrolysis through the application of nondimensionalisation techniques.

## MATERIALS AND METHODS

### Biofilm growth

**Figure 1:**
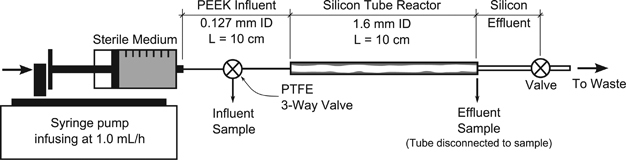
A schematic for the tube reactor assembly. Sterile LB medium is pumped through a 10 cm long, 1.6 mm inside-diameter (ID) silicon tube with biofilm growing on the walls. Small ID influent tubing was used to connect the syringe to the tube reactor. A three-way valve was used to take influent samples and the downstream end of the tube reactor was disconnected to take effluent samples.

*Escherichia coli* MJK2 (ref. 12) biofilms were grown in 10 cm long, size 14 silicon tubes (Masterflex, Cole-Parmer, IL, USA) with influent urea concentrations ranging from 0.011 to 0.221 mol/l (0.63 to 13.3 g/l) at room temperature (approximately 20 °C). *E. coli* MJK2 possesses a pJN105 plasmid that has been modified to contain the urease operon from *E. coli* DH5α(pURE14.8) and also carries a mutant chromosomal *gfp* (green fluorescent protein gene).^13,14^ Experiments were initiated by the injection of inoculum into an autoclaved reactor assembly and allowing cells to attach for 1 h. See the Supplementary Online Material for strain details and preparation of the inoculum. Flow was then started at 1.0 ml/h (Reynolds number (Re) of 0.1 in a clean tube) and maintained at 1.0 ml/min for 10 days with a KDS220 multichannel syringe pump (KD Scientific, Holliston, MA, USA). Flow was only stopped for short periods to exchange sterile 60 ml plastic syringes filled with sterile medium. The silicon tube was connected to the syringe via small-inside-diameter (0.127 mm) PEEK tubing (see Figure 1). The small-inside-diameter tubing is intended to minimise residence time and cause a high shear environment that is not conducive to biofilm growth in the influent tubing. Without biofilm growth, the average hydraulic residence time is 4.5 s in the influent tubing and 12.1 min in the silicon tube reactor.

After 10 days of biofilm growth, three consecutive effluent samples and one influent sample were taken. The three consecutive effluent samples were taken to demonstrate steady-state behaviour. Tubes that had at least one consecutive effluent urea concentration deviating from the mean of the three samples by 25% were not used in further analysis. High variation between serial replicates indicates transient behaviour such as biofilm detachment events during sampling making the data set unsuitable for modelling. The last effluent sample taken from each tube was taken to be representative and used in all subsequent models because it was taken closest to the end of the experiment when thin section samples were prepared.

Effluent samples were taken by disconnecting the downstream end of the tube and letting media flow into a 1.5 ml microcentrifuge tube at the experimental flow rate for 1 h. The influent sample was taken last by directing flow to the sample tube by the three-way valve as shown in Figure 1. The empty microcentrifuge tubes were weighed, 100 μl of 3.8% HCl was added, weighed, sample added and weighed again. The final sample pH of approximately 1.5 is effective at stopping ureolysis because the urease enzyme has been shown to lose activity at low pH.^15^ Finally, samples were filtered (to remove cells) through a 0.2 μm pore size cellulose acetate syringe filter (VWR, Randor, PA, USA) and refrigerated for later measurement of urea concentrations via HPLC. Immediately after the liquid sample was taken, the silicon tubes were destructively sampled for the determination of biofilm thickness profiles by thin sectioning as described in section ‘Biofilm thickness measurements’.

### Biofilm thickness measurements

Immediately following the liquid sampling, the tubes were dissected to determine a biofilm thickness profile along the length of each tube. Each 10 cm tube was cut into five 2 cm-long sections. The centre 1 cm of each section was cut out and cut in half lengthwise with a surgical scalpel. The remaining liquid was carefully wicked away with tissue paper before Tissue-Tek O.C.T. Compound (Sakura Finetek Inc., Torrance, CA, USA) was dispensed onto the cross-section of each tube and placed on dry ice to freeze. Once the O.C.T. was fully frozen, the cross-section of tube was peeled away leaving the biofilm embedded in the O.C.T. Microscopic investigation showed that no significant biofilm was left behind on the tube. The frozen and embedded biofilm was then completely embedded in O.C.T. in a tissue cryofixation mould. The frozen samples were stored at −20 °C for subsequent thin sectioning.

The frozen biofilm samples were cut into 5 μm-thick cross-sections and mounted in a Leica CM1850 cryostat. Sections were mounted onto charged microscopy slides (Fisherbrand Superfrost Plus Stain, Fisher Scientific, Hampton, NH, USA) and air-dried. Five sections were taken for each segment of tube. The green fluorescent protein produced by the bacteria was imaged via epifluorescence microscopy. Thin sections were imaged on a Nikon E-800 microscope equipped with a CoolSNAP MYO CCD camera (Photometrics, Tucson, AZ, USA), PhotoFluor LM-75 light source (89 North Inc., Burlington, VT, USA), Nikon Plan Apo 10X/0.45 DIC L ∞/0.17 WD 4.0 objective and a FITC filter cube (EX 480/30, DM 505 LP, EM 535/40). The raw 16-bit images were 1,940×1,460 pixels with a pixel size of 0.4499 μm. All the fluorescence images were obtained with an exposure time of 2 s.

**Figure 2:**
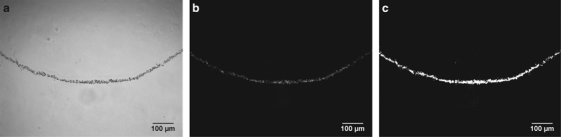
A representative example of a thin section image. (**a**) A transmitted light image shows a qualitative representation of the biofilm. (**b**) The fluorescence image, showing the green fluorescent protein (GFP) signal, is used for quantification. (**c**) The GFP fluorescence image is thresholded to differentiate between biofilm (white) and background (black), forming a binary image. The area of the biofilm signal is quantified and divided by the calculated visible arc length (0.923 mm) to calculate a representative average biofilm thickness.

Raw images were thresholded in the open source software FIJI^16^ using the automatic triangle method.^17^ The triangle method proved to provide the most representative results over other commonly used methods such as Otsu (1979). The thresholded images were quantified to determine average biofilm thicknesses (L_f_). This was done by dividing the calculated arc length of the section of tube shown in the image (0.923 mm, see Supplementary Table SI) by the area of the biofilm cross-section that was obtained from the thresholded image.^18^ Images that contained significant defects were excluded from the analysis. Defects included dust particles, bubbles and geometric irregularities that would cause inaccuracy in the thickness measurements. Average thickness from all usable replicates are presented (see Supplementary Tables SIII–SX for thickness calculations and raw data). Images were not taken when samples did not contain any visible biofilm and the thickness was recorded as zero. Figure 2 shows a typical thin section and the analysis that was performed on it.

### Urea quantification

Urea was analysed using high pressure liquid chromatography (HPLC) with in-autosampler derivatisation.^12,19^

### Urea hydrolysis rate determination

**Figure 3:**
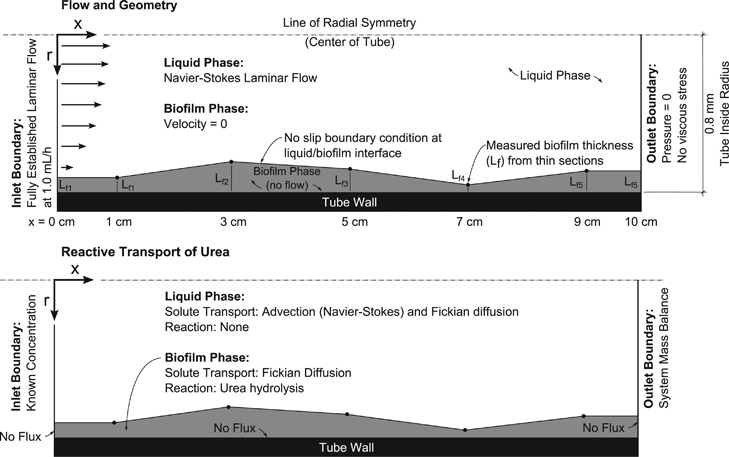
COMSOL model overview with boundary conditions.

Tube reactors were modelled using COMSOL Multiphysics version 4.3a (COMSOL Inc., Burlington, MA, USA) to fit kinetic parameters to the measured urea consumption over the length of the tube reactors. The tube reactors were modelled as a two-dimensional rotated axisymmetric geometry. Navier–Stokes equations were solved to obtain a flow field and it was assumed that there is zero fluid flow in the biofilm domain. The thin section data were used to reconstruct the biofilm profile for each tube reactor. It was assumed that the biofilm thickness was constant for discrete values of *x* (see Figure 3 for model coordinates) and the thickness was linearly interpolated between measured points. Biofilm thickness could not be measured immediately at the influent or effluent of the reactors (*x*=0 and 10 cm) because of potential artifacts owing to the removal of the silicon tubes from the experimental system, so it was assumed that *L*_f_ at *x*=0 cm equaled *L*_f_ at *x*=1 cm and *L*_f_ at *x*=10 cm equaled *L*_f_ at *x*=9 cm. Urea transport was calculated with advection by the Navier–Stokes flow field and Fickian diffusion. No reduction in diffusive transport in the biofilm domain was considered so diffusion characteristics were assumed to be constant throughout the simulation. Preliminary analysis showed that implementing increased mass transport resistance of solutes in the biofilm as in ref. 20 affected the rate fitting minimally (<1% difference in the thickest biofilms, data not shown). Boundary conditions and modelling assumptions are indicated in Figure 3.

The urea hydrolysis reaction rate, *R*, was assumed to be constant within the entire biofilm in each tube (independent of concentration) and constrained to the biofilm domain (no reaction in the liquid phase). Justification for the constant reaction rate assumption follows in section ‘Reactive transport characterisation’. The influent and effluent urea concentrations are known so the urea hydrolysis rate was fitted such that the experimentally determined concentrations fit the model. The urea concentration at the outlet boundary is non-constant in space (that is, across the cross-section of the outlet); thus, rather than minimising the difference between modelled and experimental concentrations directly, the difference between the modelled and experimental flux of urea averaged across the tube cross-section was minimised. The last of the three effluent urea concentrations was taken to be the representative average concentration across the cross-section of the tube because it was taken closest to the time of destructive sampling. The known effluent urea flux, *J*_EF_, can be calculated by multiplying the effluent concentration, *C*_EF_, by the volumetric flow rate, *Q* such that

(2)JEF=CEFQ.

*C*_EF_ is an average and well-mixed value. The modelled total effluent urea flux, *J*’_EF_, was calculated by integrating over the area of the liquid phase at the effluent such that

(3)J'EF=2π∫0ID/2−LfCurdr,

where *u* is the *x* component of the fluid velocity, *C* is the local urea concentration and ID is the tube inside diameter (1.6 mm). The model was run iteratively where the biofilm ureolysis rate was varied such that the sum of the squared error between *J*_EF_ and *J*′_EF_ is minimised. The COMSOL optimisation module was used to find a urea hydrolysis rate, *R*, that minimises the squared error, (*J*_EF_−*J*’_EF_)^2^, using the Nelder–Mead method^21,22^ with a nondimensional fitting tolerance of 10^−6^.

Fitted rate values for each tube were compiled into a data set that represents reaction rates at a range of urea concentrations. An average urea concentration within the biofilm volume, *C*_Urea,BF_, was calculated for each tube and was taken to be the representative urea concentration corresponding to the rate that was fitted. A Michaelis–Menten (M–M) rate, *R*_m–m_, relationship was fitted to the compiled data using the least squares curve fitting tool (cftool) in MATLAB R2012a (The MathWorks, Inc., Natick, MA, USA):

(4)Rm–m=RmaxCkm+C.

*R*_max_ is the maximum ureolysis rate and *k*_m_ is a half saturation coefficient. It was theorised that the M–M relationship would fit the data best but other rate relationships were fit for comparison. For a reaction of order, *n*, with respect to urea concentration a generalised rate law can be written as

(5)Rn=knCn.

First-order (*n*=1, linear) and zero-order (*n*=0, constant rate) relationships have been used previously to describe microbial ureolysis^7,12,23,24^ so they were also included in the regression analysis.

## RESULTS AND DISCUSSION

### Analytical results

#### Urea measurement

Measureable urea hydrolysis was observed in all tube reactors after 10 days of operation. Influent urea concentrations varied between 0.011 and 0.221 mol/l. Effluent concentrations varied between 0.003 and 0.208 mol/l including all serial replicates. Tubes 5, 10 and 11 were eliminated from the analysis because the steady-state condition was not met (>25% deviation from the mean). The reader is referred to the Supplementary Table SII for all urea measurements.

#### Biofilm thickness profiles

Biofilm profiles were successfully obtained from 11 tube reactor runs. Biofilm thicknesses ranged from 0.6 to 222.0 μm with an average across all observations of 18.7 μm. Assuming zero flow through the biofilm, laminar flow is expected in all tube reactors (a maximum Re=0.18 was calculated for the reactor with the thickest biofilm observed).

**Figure 4:**
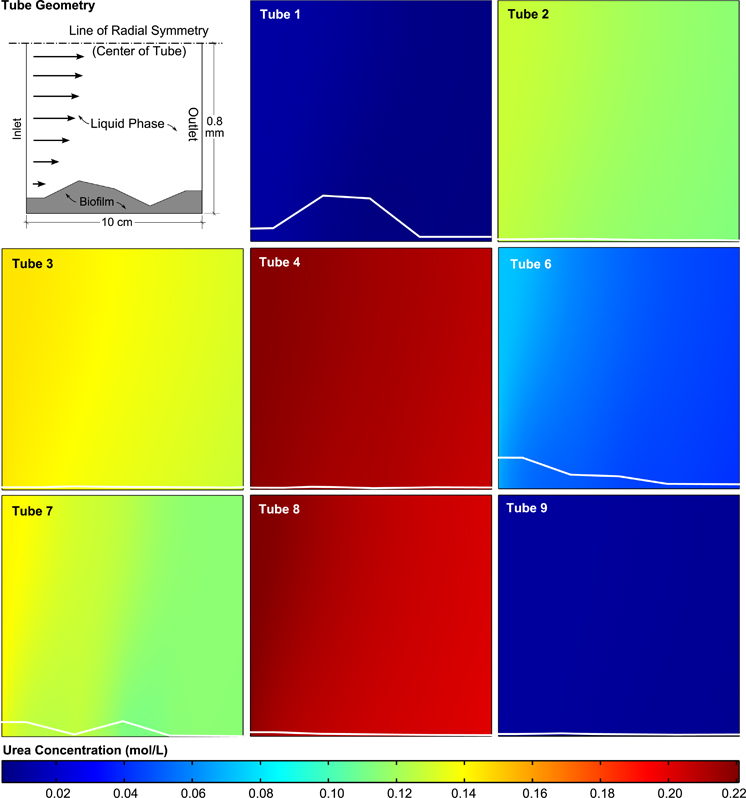
Urea concentration as predicted by the reactive transport model for each tube reactor used in the analysis. The concentration map for all the tubes is plotted using the same colour scale making it evident that each tube had a different but within each tube very narrow urea concentration range. Biofilm profiles for each tube obtained from microscopy data are shown as white lines in each panel. Note the very slight concentration gradients radially outward from the centre of the tubes.

Five replicate biofilm thickness measurements were collected for each profile point. 5.5% of the measurements were eliminated from the analysis due to apparent irregularities such as bubbles in the cutting medium, dust particles and deformed sections. All biofilm thickness measurements presented were at least measured in duplicate with an average of 4.7 measurements per thickness value. Raw biofilm measurements can be found in the Supplementary Online Material and the average profiles used in the model can be found in Figure 4.

#### Sources of error

There are some distinct sources of error associated with the tube reactor experiment that must be addressed. The most significant source of error is expected to be in defining the biofilm thickness profile. If the biofilm thickness throughout the tube is not well characterised, the calculated rates are not accurate because the rate fitting implicitly depends on the amount of biofilm present. There are five potential sources of error for the estimation of the biofilm thickness profile: (i) biofilm at the influent and effluent of the tubes cannot be accurately quantified, (ii) biofilm thickness between measurement points may not be well approximated by linear interpolation, (iii) biofilm thickness may not be constant around the entire cross-section of the tube and (iv) significant detachment events have the potential to occur during sampling for aqueous analysis.

In the first three potential sources of error, the problem is that the entire tube, and biofilm within that tube, cannot reasonably be imaged in three dimensions. These errors are directly related to the lack of the ability to sample at a high spatial resolution and can be considered random errors. Random errors in this system can be expected to have the same likelihood for overestimation as for underestimation. In other words, the sampling regime is just as likely to miss a thick area of biofilm as a thin area. Although more advanced techniques would be required, the low spatial resolution problem can be resolved. Three-dimensional imaging techniques such as X-ray microtomography^25–27^ and nuclear magnetic resonance imaging^28,29^ have the potential to image simple biofilm systems and better link biofilm geometry to effective reaction rate estimations.

Error associated with detachment events during the sampling process cannot be quantified here but the sign of the error and its effects is known. If a large detachment event were to happen during aqueous sampling or between sampling and thin sectioning, the biofilm profile during aqueous sampling would be unknown but it had to be thicker than would be shown in the thin sections. This would always lead to an overestimation of the effective reaction rate in that particular tube due to the underestimation of biofilm volume. Similarly to the other class of random errors, this problem could be minimised through the use of more advanced three-dimensional imaging techniques. These more advanced techniques would still need to be utilised carefully as to not disturb the biofilm.

There is another potential source of error that is not related to the estimation of biofilm thickness. This relates to the assumption that the biofilm is homogeneous. There could be differences in cell density, enzyme production (urease in this case) or general metabolic activity that could cause inaccuracies when estimating rate constants. This possibility was not investigated directly in this study but its influence cannot be ruled out and should be the focus of future work. Systems with electron donor or acceptor limitation would be particularly interesting for such future studies.

### Reactive transport characterisation

The Damköhler number (Da) is defined as the time scale of convective transport divided by the time scale of reaction within a control volume.^30^ For a first-order reaction, the Damköhler number can be defined as

(6)Da=k1τ,

where *τ* is the average fluid retention time. This standard expression for Da requires modification for a system with rates defined as occurring only in the biofilm phase. Equation (6) assumes that reaction is occurring within the entire volume for which *τ* is calculated (the liquid volume). In this case, the reaction is not occurring in the liquid volume so the transport rate and reaction rates must be normalised for the volumes in which they occur.

Similarly, the Péclet number (Pe) can be defined as the ratio of the advection rate of a solute to the diffusion rate of the same solute.^31^ The Péclet number can be expressed as

(7)Pe=vLD,

where *D* is the diffusion coefficient, which is 1.38×10^−9^ m^2^/s for urea in pure water at 25 °C (ref. 31), *v* is an average fluid velocity and *L* is a representative length scale. The values of *v* and *L* can be chosen based on what is being characterised in the system. In this case, both axial and radial behaviour is of interest so two Péclet numbers are defined. The first, Pe_*x*_, characterises axial behaviour where the velocity is taken to be the average magnitude of the *x* component of velocity, *v*_*x*_. The second, Pe_r_, characterises radial behaviour where the velocity is taken to be the average magnitude of the *r* component of velocity, *v*_*r*_. The length scales are different for radial and axial behaviour. The tube length, *l*_tube_, is the length scale for axial flow and the tube diameter *d*_tube_ is the length scale for radial flow such that

(8)Pex=vxltubeD,

and

(9)Per=vrdtubeD.

The last dimensionless parameter considered is the Thiele modulus (*ϕ*) which is the ratio of the time scale of reaction to the time scale of diffusion. The Thiele modulus is simple to calculate for first-order reactions.^31^

(10)ϕ2=k1L2D.

The length scale (*L*) here is a biofilm thickness (*L*_f_) because the Thiele modulus, in this case, is calculated as a metric to quantify potential diffusion limitation of the biofilms in this system. Biofilm thickness is not constant in these systems so average high and low values for each tube are used to bound overall behaviour. Table 1 shows the calculated dimensionless parameters (average, minimum and maximum) for each tube reactor in the study. The values used in the calculation of the dimensionless parameters were taken from the finite element model described in section ‘Kinetic parameter fitting’

In general, the dimensionless parameter analysis shows that advection dominates the system (rather than diffusive transport and urea hydrolysis) and that the biofilms are not diffusion limited. The Damköhler number shows that, for this particular system, the time scale of advective transport is larger than the time scale of reaction. Axial Péclet numbers are all much greater than one (Pe_*x*_ ≫ 1) indicating that the system is strongly advection dominated. Only a small fraction of axial urea transport can be contributed to diffusion. The radial Péclet number indicates advective and diffusive urea transport are more balanced with diffusive and advective transport contributing more equally in the radial direction.

There are two primary characteristics that control Pe_*r*_ assuming a constant diffusion coefficient. First, the bulk flow rate affects Pe_*r*_; a higher overall system flow rate will translate into greater overall velocities, including radial velocities. Second, the heterogeneity of the biofilm profile directly affects radial velocities; as the biofilm profile becomes more constant (that is, the closest to *L*_f_ being equal at all values of *x*), the average radial velocity will decrease, thus decreasing Pe_*r*_. It can also be expected that flow rate and biofilm thickness heterogeneity could be linked. Although this physiological relationship was not studied here it has been shown that biofilms adapt to their shear and solute transport environments.^32,33^ *E. coli* biofilms have been shown to adapt their architecture to specific hydrodynamic and nutrient conditions. Specifically, when nutrients are provided in excess (as is expected in this study due to high Da and low ϕ values) *E. coli* biofilms have been shown to adapt to resist shear stress.^34^

The Thiele modulus was the most variable dimensionless parameter in the system but all values were found to be less than one indicating that the time scale for diffusion is generally shorter than the time scale of reaction. This means that urea hydrolysis within biofilms in this study are not strongly diffusion limited. In other words, the bulk liquid concentrations are expected to be approximately equal to the concentrations found within the biofilm (that is, no steep gradients in urea concentration). This finding is an important validation for the approach that was taken in determining kinetic rate constants where the reaction rate and the corresponding biofilm urea concentration (*C*_Urea,BF_) in each tube was assumed to be constant. Visual evidence for small Thiele modulus behaviour can also be found in Figure 4 where the urea concentration does not appear to change significantly within the biofilms relative to the bulk fluid.

### Kinetic parameter fitting

Tube reactors that met the steady state criteria were modelled using individual finite element models, and the urea hydrolysis rate, *R*, was varied until the differences between the modelled and experimental urea fluxes were minimised. The representative concentration corresponding to the calculated reaction rate was assumed to be the average urea concentration within the biofilm volume of each tube.

**Figure 5:**
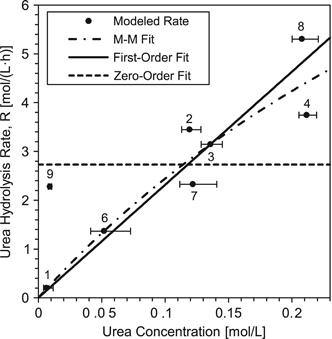
Average urea concentration within the biofilm volume versus estimated urea hydrolysis rate. The modeled points correspond to the rate calculated by the finite element models versus the average urea concentration in the biofilm volume, *C*_Urea,BF_. Error bars represent the range of urea concentration within the biofilm volume as estimated by the finite element models. Points are labeled with the tube reactor numbers from which they were obtained (see for example Figure 4).

The resulting data from the finite element models allowed for regression analysis considering Michaelis–Menten (M–M), first- and zero-order rate models. A plot of urea hydrolysis reaction rate versus representative concentration along with rate model fits can be found in Figure 5. M–M and first-order models fit the data best (root-mean-square error of 1.01 and 0.97 mol/(l h) respectively), whereas the zero order was a poor fit compared with the others (root-mean-square error=1.56 mol/(l h)). M–M and first-order model fits are not statistically different at 95% confidence over the range of concentrations considered in this study. Rate constants for the M–M model were estimated to be *k*_m_=0.55 mol/l and *r*_max_=15.9 mol/(l h) and for the first-order model *k*_1_=23.2±6.2 h^−1^ (± is the 95% confidence interval).

The demonstration of first-order behaviour is important for modelling more complex systems and is a significant finding in this work. Others have used first-order kinetics for ureolysis systems involving ureolysis-driven mineralisation;^7,23^ however, the applicability of that model had remained undemonstrated for any biofilm system until now. For reactions obeying M–M kinetics, as has been shown for urea hydrolysis,^11,15,35^ it is typically assumed that first-order kinetics should only be applied to systems where the reaction substrate is at low concentration (C ≪ *k*_m_). The results shown here indicate that published kinetic values from pure enzyme or planktonic biological experiments may not be appropriate for use in systems where attached cells and biofilms are the primary catalyst. Diffusion limitation is routinely cited as the reason for different kinetic behaviour in biofilms but the results of this work suggest that kinetic differences can occur even without evidence for significant diffusional mass transport limitations.

The mass transport limitation of urea was considered in this analysis independent of other solutes. As an example, oxygen limitation could also be investigated since biofilms in nature are often at least partially oxygen-limited. In this study, it is not expected that oxygen would be limiting due to the high gas permeability of the silicon tubing used, but in natural systems oxygen may be depleted which could impact the kinetics of urea hydrolysis. As a result, mass transport limitations of other solutes may need to be considered in other systems when applying the approach outlined in this study.

### Practical applications

The study presented has direct and indirect applications. The most direct application of this work is the continuation of laboratory studies using *E. coli* MJK2. The biofilm-specific kinetic parameters obtained in this study can be used in conjunction with tools such as confocal microscopy, flow cells and finite element modelling in more complex systems to further investigate local chemical gradients within ureolytic biofilms. Systems that would be particularly well suited include ureteral catheters^1^ and urea-hydrolysis-driven microbial carbonate precipitation.^3,4^

Beyond urea hydrolysis systems specifically, this paper presents a robust method for the systematic characterisation of effective reaction rates in biofilms in flow systems. The small scale of the tube reactor method has advantages over more common laboratory bench-scale biofilm reactors such as the CDC reactor,^36^ annular reactors^37^ or drip-flow reactors.^38^ The main advantages relate to the cost and the reduction of waste. The simple tube reactor requires no specialised equipment other than a syringe pump or a similar pump that provides a constant and slow flow. The shear environment can also be easily tailored to the specific requirements of the study by varying the flow rate or tube size. The tube reactor method is also well suited for studies where hazardous materials are used. Studies focusing on quantifying the degradation of hazardous substances by biofilms may find use in small tube reactor studies because experiments could be designed that produce a minimal amount of hazardous waste (for example, chlorinated aromatics, hydrocarbons, nitroaromatics and pharmaceuticals). Studies where high cost substrates are used (for example, stable isotope compounds) may also find use in the tube reactor system. There are also disadvantages to the tube reactor method as compared with larger volume approaches. Some studies may require larger sample volumes for multiple analyses, making the use of such a low-flow-rate system impractical.

The particular system that was analysed in this work was found to be not diffusion limited and the concentration range for each tube was small. In this case, a much simpler calculation could have been performed to determine the effective volumetric reaction rate in the biofilm reactor. When the concentration in the entire biofilm volume can be considered constant an average reaction rate in the biofilm in each tube could be determined by simply calculating the biofilm volume and dividing it by the difference in concentration across the tube. The constant concentration assumption cannot be made for all systems so the more rigorous inverse modelling approach was presented here. In general, if larger molecules with lower diffusion coefficients are the subject of a kinetic analysis or if thicker biofilms are present, the more rigorous method would still be required.

### Conclusions

The determination of biofilm-specific reaction rates is important for the accurate micro-scale modelling of biofilm systems. In this work, biofilm-specific urea hydrolysis rate coefficients were determined (both Michaelis–Menten and first-order rate models) in a tube reactor system. It was found that for the chemical and hydrodynamic conditions present in this study, a first-order (linear) rate model fits the reaction rate versus concentration data as well as the Michaelis–Menten model which is more difficult to work with computationally. This work along with the previous characterisation of *E. coli* MJK2 in planktonic culture makes the organism a valuable tool for the study of various aspects of ureolysis in biofilms. Applications include medical, environmental science and engineering research topics. Beyond the specific findings related to ureolysis, the method presented in this work has wider applications to other biofilms where a biofilm-specific reaction rate needs to be determined.

## Figures and Tables

**Table 1:** Average dimensionless parameters for all tube reactors along with their minima (Min) and maxima (Max)

	*Da*	*Pe*_x_	*Pe*_r_	Φ
Average	4.42	1.42×10^4^	2.31	4.75×10^−2^
Min	3.85	1.34×10^4^	1.00	1.63×10^−3^
Max	4.64	1.64×10^4^	6.00	3.36×10^−1^

Da and Pe are calculated for each tube and **Φ** is calculated for all *L*_f_ measurements.
