# Supplementary material for: Estimation of a biofilm-specific reaction rate: kinetics of bacterial urea hydrolysis in a biofilm
Source: NPJ Biofilms Microbiomes. 2015 Sep 16;1:15014–. doi: 10.1038/npjbiofilms.2015.14 (PMC5515221; doi:10.1038/npjbiofilms.2015.14)
Supplement: Supplementary Information [file npjbiofilms201514-s1.pdf]

SUPPLEMENTAL ONLINE MATERIAL for:

Estimation of biofilm-specific reaction rates with application to bacterial urea hydrolysis

Authors

James M. Connolly [a,b]	james.m.connolly@gmail.com
Benjamin Jackson [a,c]	jackson@math.montana.edu
Adam P. Rothman [a,b]	Adam.P.Rothman@tsocorp.com
Isaac Klapper [d]	klapper@temple.edu
Robin Gerlach [a,b,*]	robin_g@biofilm.montana.edu

Author Affiliations

[a] Center for Biofilm Engineering, Montana State University, 366 EPS Building, P.O. Box 173980, Montana State University, Bozeman, MT 59717-3980, USA.

[b] Department of Chemical and Biological Engineering, Montana State University, 306 Cobleigh Hall, P.O. Box 173920, Bozeman, MT 59717-3920, USA.

[c] Department of Mathematical Sciences, Montana State University, P.O. Box 172400, Bozeman, MT 59717-2400, USA.

[d] Department of Mathematics, Temple University, 1805 N Broad Street, Philadelphia, PA 19122-6094

* Corresponding author: Robin Gerlach: Tel.: +1 (406) 994-1840

Funding

The authors were funded by the National Science Foundation through NSF award No. DMS-0934696 and by the U.S. Department of Energy Grant Numbers DE-FG-02-09ER64758, DE-FE0004478, DE-FE0009599 and DE-FG02-13ER86571. J.M.C was also supported by a NSF-IGERT fellowship in Geobiological Systems at Montana State University (DGE-0654336).

Strain and growth conditions

Escherichia coli MJK2 (Connolly et al., 2013) was used as the model biofilm-producing ureolytically active organism in this study. MJK2 possesses a pJN105 plasmid that has been modified to contain the urease operon from *E. coli* DH5α(pURE14.8) (Collins and Falkow, 1988). The urease-carrying plasmid, pMK001, contains an L-arabinose-inducible promoter and encodes for gentamycin resistance. MJK2 also possesses a mutant chromosomal *gfp* (green fluorescent protein gene) variant that can be used for imaging. The parent strain of MJK2 is *E. coli* AF504 which is a nalidixic acid resistant derivative of *E.coli* K12 strain MG1655 (Folkesson et al., 2008). The growth medium for MJK2 was Luria–Bertani (LB) medium containing 10 g/L tryptone, 5 g/L yeast extract, 10 g/L NaCl, supplemented with 50 mM (7.5 g/L) L-arabinose, 10 µM $NiCl_2$, 10 µg/mL gentamycin and varying amounts of urea. The medium was adjusted to a pH of 6 with HCl.

100 mL liquid cultures were inoculated from frozen stock cultures at a concentration of 1.0 µL per mL in 250 mL Erlenmeyer flasks at 37ºC on horizontal shakers running at approximately 150 rpm. The urea concentration of the starter culture matched the experimental concentration in the tube reactors. 100 µL of culture was transferred into 100 mL of the same media after approximately 24 hours. Cells from the transfer culture were harvested after approximately 12 hours by centrifugation at 4200×g for 10 minutes at 5ºC in 50 mL conical centrifuge tubes containing 40 mL of culture. Cells were suspended in sterile phosphate buffered saline (PBS) by vigorous vortexing. Cells were washed one more time by centrifugation and resuspension. PBS had final concentrations of 8 g/L NaCl, 0.61 g/L KH_2PO_4, 0.96 g/L K_2HPO_4 and was adjusted to a pH of 7 with HCl. The cell suspension was adjusted to an optical density of 0.6 by diluting with additional PBS after the final cell wash. Optical density was measured on triplicate 100 µL

samples in polycarbonate 96-well plates (light path length of 0.26 cm) with a BioTek Instuments (Winooski, VT, USA) Synergy HT Multi-Mode Microplate Reader, and the data were analyzed using Gen5 software. The cell suspensions were used as the inoculum for the biofilm growth experiments.

References relating to the strain and growth conditions:

Collins CM, Falkow S. 1988. Genetic Analysis of an *Escherichia coli* Urease Locus: Evidence of DNA Rearrangement. *Journal of Bacteriology* 170:1041–1045.

Connolly J, Kaufman M, Rothman A, Gupta R, Redden G, Schuster M, Colwell F, Gerlach R. 2013. Construction of two ureolytic model organisms for the study of microbially induced calcium carbonate precipitation. *Journal of Microbiological Methods* 94:290–299.

Folkesson A, Haagensen JAJ, Zampaloni C, Sternberg C, Molin S. 2008. Biofilm Induced Tolerance towards Antimicrobial Peptides. *Plos One* 3:e1891.

Biofilm Thin Section Calculations

The following calculations were applied to the thresholded thin section images (e.g. Figure 2C) in order to estimate a representative biofilm thickness at discrete points within the tube.

Table SI – Constants used in thickness calculations

Pixel Size (p)	0.449965 µm/px
Image Size (X by Y)	1940 by 1460 px
Total Image Area (A)	573471.78 μm^2
Visible Biofilm Arc Length (S)	922.95 µm
Tube Inside Radius (r)	800 µm

The visible biofilm arc length was calculated by first calculating the known chord length (C) of the visible biofilm arc,

$$C = X\,p = 872.61\,\mu m\,. \qquad \text{(S1)}$$

Images were taken such that they were centered in the long dimension (X) of the image. The central angle of the biofilm arc (θ) can then be calculated with a simple trigonometric relationship where

$$\theta = 2\sin^{-1}\left(\frac{0.5\,C}{r}\right) = 1.154\,\text{rad}\,, \qquad \text{(S2)}$$

and finally the biofilm arc length can be calculated by

$$S = r\,\theta = 922.95\,\mu m\,. \qquad \text{(S3)}$$

Images were thresholded as stated in the main article and the image area occupied by biofilm (A_f) was determined in ImageJ. The calculated average biofilm thickness (L_f) for each image, as reported in Tables S2-S9, was then be calculated by

$$L_f = {A_f}/{S}\,. \qquad \text{(S4)}$$

It should be noted that this calculation is only valid for thin biofilms. Thin in this context means that the biofilm thickness is much smaller than the tube radius ($L_f << r$). If the thin condition is not met, the visible area of the biofilm will be dependent on L_f due to the vertically (rather than radially) cut off biofilms at the edges of the image.

Urea Measurements

Table SII – Urea measurements obtained in the study including those that were eliminated from the analysis.

Tube #	Sample		Concentration (mol/L)	Effluent Mean (mol/L)	Variation from Mean	Notes
1	1	Out A	0.00368	0.00422	12.80%	
	1	Out B	0.00438		-3.76%	
	1	Out C	0.00461		-9.04%	
	1	In	0.01168			
2	2	Out A	0.10189	0.10872	6.29%	
	2	Out B	0.10972		-0.92%	
	2	Out C	0.11456		-5.37%	
	2	In	0.12835			
3	3	Out A	0.13588	0.13367	-1.65%	
	3	Out B	0.13435		-0.50%	
	3	Out C	0.13079		2.15%	
	3	In	0.14519			
4	4	Out A	0.20390	0.20637	1.20%	
	4	Out B	0.20741		-0.50%	
	4	Out C	0.20781		-0.70%	
	4	In	0.21952			
5	5	Out A	0.00328	0.00460	28.70%	Excluded due to high effluent variation (> 25%)
	5	Out B	0.00311		32.43%	
	5	Out C	0.00741		-61.13%	
	5	In	0.01220			
6	6	Out A	0.04851	0.04632	-4.72%	
	6	Out B	0.04734		-2.19%	
	6	Out C	0.04312		6.91%	
	6	In	0.07271			
7	7	Out A	0.11966	0.11868	-0.83%	
	7	Out B	0.12087		-1.84%	
	7	Out C	0.11550		2.68%	
	7	In	0.14066			
8	8	Out A	0.19859	0.20216	1.77%	
	8	Out B	0.20585		-1.82%	
	8	Out C	0.20205		0.06%	
	8	In	0.22077			
9	9	Out A	0.00653	0.00722	9.54%	
	9	Out B	0.00686		5.05%	
	9	Out C	0.00828		-14.59%	
	9	In	0.01053			
10	10	Out A	0.03980	0.04933	19.31%	Excluded due to high effluent variation (> 25%)
	10	Out B	0.03969		19.53%	
	10	Out C	0.06848		-38.84%	
	10	In	0.08295			
11	11	Out A	0.08498	0.09962	14.70%	Excluded due to high effluent variation (> 25%)
	11	Out B	0.13257		-33.08%	
	11	Out C	0.08131		18.38%	
	11	In	0.15836			

Note: Samples “Out C” and “In” were taken as the representative concentrations for each tube.

Biofilm Thin Section Data

Table SIII - Tube 1 thin section data.

Tube #	x Distance from Inlet	Calculated Thickness (L_f)	Averages from Replicates: x Distance from Inlet	Averages from Replicates: Calculated Thickness(L_f)
	[cm]	[µm]	[cm]	[µm]
1	1	50.07	1	45.74
1	1	44.72	3	155.32
1	1	40.14	5	143.15
1	1	45.63	7	14.90
1	1	48.12	9	16.18
1	3	173.34		
1	3	159.22		
1	3	144.21		
1	3	144.51		
1	5	62.61		
1	5	136.96		
1	5	132.68		
1	5	161.45		
1	5	222.03		
1	7	17.00		
1	7	11.74		
1	7	13.45		
1	7	17.40		
1	9	12.94		
1	9	14.66		
1	9	20.94		

Table SIV - Tube 2 thin section data.

Tube #	x Distance from Inlet	Calculated Thickness (L_f)	Averages from Replicates: x Distance from Inlet	Averages from Replicates: Calculated Thickness (L_f)
	[cm]	[μm]	[cm]	[μm]
2	1	9.22	1	9.08
2	1	10.17	3	11.53
2	1	8.66	5	9.09
2	1	8.33	7	7.98
2	1	9.03	9	5.34
2	3	9.15		
2	3	11.42		
2	3	11.26		
2	3	14.30		
2	5	9.87		
2	5	10.47		
2	5	8.99		
2	5	7.97		
2	5	8.14		
2	7	7.56		
2	7	7.93		
2	7	8.46		
2	7	7.80		
2	7	8.14		
2	9	3.94		
2	9	5.75		
2	9	4.92		
2	9	6.51		
2	9	5.56		

Table SV - Tube 3 thin section data.

Tube #	x Distance from Inlet	Calculated Thickness (L_f)	Averages from Replicates: x Distance from Inlet	Averages from Replicates: Calculated Thickness (L_f)
	[cm]	[μm]	[cm]	[μm]
3	1	8.79	1	8.47
3	1	8.08	3	10.91
3	1	9.16	5	9.21
3	1	7.66	7	9.00
3	1	8.66	9	9.00
3	3	7.21		
3	3	10.18		
3	3	11.25		
3	3	11.48		
3	3	14.46		
3	5	9.68		
3	5	9.44		
3	5	7.93		
3	5	8.87		
3	5	10.15		
3	7	9.08		
3	7	8.94		
3	7	8.96		
3	7	9.13		
3	7	8.90		
3	9	8.02		
3	9	9.69		
3	9	9.98		
3	9	8.31		

Table SVI - Tube 4 thin section data.

Tube #	x Distance from Inlet	Calculated Thickness (L_f)	Averages from Replicates: x Distance from Inlet	Averages from Replicates: Calculated Thickness (L_f)
	[cm]	[μm]	[cm]	[μm]
4	1	5.22	1	6.25
4	1	6.29	3	8.43
4	1	6.52	5	4.43
4	1	6.01	7	6.93
4	1	7.19	9	6.65
4	3	7.45		
4	3	11.04		
4	3	7.34		
4	3	7.91		
4	3	8.43		
4	5	4.95		
4	5	3.78		
4	5	5.20		
4	5	4.14		
4	5	4.08		
4	7	7.06		
4	7	6.79		
4	9	5.65		
4	9	5.18		
4	9	9.24		
4	9	6.51		

Table SVII - Tube 6 thin section data.

Tube #	x Distance from Inlet	Calculated Thickness (L_f)	Averages from Replicates: x Distance from Inlet	Averages from Replicates: Calculated Thickness (L_f)
	[cm]	[μm]	[cm]	[μm]
6	1	160.56	1	101.85
6	1	78.07	3	47.97
6	1	121.03	5	40.93
6	1	75.33	7	7.42
6	1	74.29	9	17.09
6	3	54.88		
6	3	41.51		
6	3	47.37		
6	3	43.13		
6	3	52.98		
6	5	30.17		
6	5	36.47		
6	5	49.37		
6	5	43.09		
6	5	45.54		
6	7	7.90		
6	7	7.06		
6	7	6.56		
6	7	7.40		
6	7	8.20		
6	9	18.24		
6	9	19.96		
6	9	15.88		
6	9	17.07		
6	9	14.28		

Table SVIII - Tube 7 thin section data.

Tube #	x Distance from Inlet	Calculated Thickness (L_f)	Averages from Replicates: x Distance from Inlet	Averages from Replicates: Calculated Thickness (L_f)
	[cm]	[μm]	[cm]	[μm]
7	1	63.97	1	48.73
7	1	45.92	3	8.13
7	1	45.70	5	51.94
7	1	42.85	7	3.63
7	1	45.18	9	1.26
7	3	7.56		
7	3	7.44		
7	3	7.49		
7	3	10.02		
7	5	52.18		
7	5	43.98		
7	5	49.63		
7	5	61.33		
7	5	52.60		
7	7	3.19		
7	7	3.86		
7	7	2.86		
7	7	2.29		
7	7	5.93		
7	9	1.06		
7	9	1.35		
7	9	1.26		
7	9	1.38		
7	9	1.04		

Table SIX - Tube 8 thin section data.

Tube #	x Distance from Inlet	Calculated Thickness (L_f)	Averages from Replicates: x Distance from Inlet	Averages from Replicates: Calculated Thickness (L_f)
	[cm]	[μm]	[cm]	[μm]
8	1	12.01	1	13.34
8	1	14.55	3	8.73
8	1	13.86	5	6.23
8	1	13.92	7	11.04
8	1	12.36	9	3.85
8	3	8.08		
8	3	9.26		
8	3	7.74		
8	3	8.59		
8	3	9.99		
8	5	5.84		
8	5	5.72		
8	5	7.12		
8	7	11.77		
8	7	10.75		
8	7	10.17		
8	7	9.67		
8	7	12.83		
8	9	3.50		
8	9	4.50		
8	9	3.29		
8	9	3.09		
8	9	4.86		

Table SX - Tube 9 thin section data.

Tube #	x Distance from Inlet	Calculated Thickness (L_f)	Averages from Replicates: x Distance from Inlet	Averages from Replicates: Calculated Thickness (L_f)
	[cm]	[μm]	[cm]	[μm]
9	1	0.62	1	0.76
9	1	0.65	3	2.00
9	1	0.92	5	2.84
9	1	0.80	7	2.21
9	1	0.80	9	2.15
9	3	2.06		
9	3	2.00		
9	3	2.85		
9	3	1.47		
9	3	1.64		
9	5	2.75		
9	5	2.99		
9	5	3.40		
9	5	1.98		
9	5	3.11		
9	7	1.35		
9	7	1.90		
9	7	2.44		
9	7	2.10		
9	7	3.27		
9	9	2.14		
9	9	2.53		
9	9	2.08		
9	9	1.87		
